# Pre-enrichment-free detection of hepatocellular carcinoma-specific ctDNA via PDMS and MEMS-based microfluidic sensor

**DOI:** 10.1007/s00604-024-06315-2

**Published:** 2024-04-02

**Authors:** Zeynep Çağlayan Arslan, Meltem Okan, Haluk Külah

**Affiliations:** 1 Department of Electrical and Electronics Engineering, METU, Ankara, Turkey; 2 Department of Micro and Nanotechnology, METU, Ankara, Turkey; 3 METU MEMS Research and Application Center, Ankara, Turkey

**Keywords:** Microfluidic MEMS chip, Electrochemical sensor, ctDNA detection, HCC, PDMS-based microfluidics

## Abstract

The growing interest in microfluidic biosensors has led to improvements in the analytical performance of various sensing mechanisms. Although various sensors can be integrated with microfluidics, electrochemical ones have been most commonly employed due to their ease of miniaturization, integration ability, and low cost, making them an established point-of-care diagnostic method. This concept can be easily adapted to the detection of biomarkers specific to certain cancer types. Pathological profiling of hepatocellular carcinoma (HCC) is heterogeneous and rather complex, and biopsy samples contain limited information regarding the tumor and do not reflect its heterogeneity. Circulating tumor DNAs (ctDNAs), which can contain information regarding cancer characteristics, have been studied tremendously since liquid biopsy emerged as a new diagnostic method. Recent improvements in the accuracy and sensitivity of ctDNA determination also paved the way for genotyping of somatic genomic alterations. In this study, three-electrode (Au-Pt–Ag) glass chips were fabricated and combined with polydimethylsiloxane (PDMS) microchannels to establish an electrochemical microfluidic sensor for detecting c.747G > T hotspot mutations in the TP53 gene of ctDNAs from HCC. The preparation and analysis times of the constructed sensor were as short as 2 h in total, and a relatively high flow rate of 30 µl/min was used during immobilization and hybridization steps. To the best of our knowledge, this is the first time a PDMS-based microfluidic electrochemical sensor has been developed to target HCC ctDNAs. The system exhibited a limit of detection (LOD) of 24.1 fM within the tested range of 2–200 fM. The sensor demonstrated high specificity in tests conducted with fully noncomplementary and one-base mismatched target sequences. The developed platform is promising for detecting HCC-specific ctDNA at very low concentrations without requiring pre-enrichment steps.

**Graphical Abstract:**

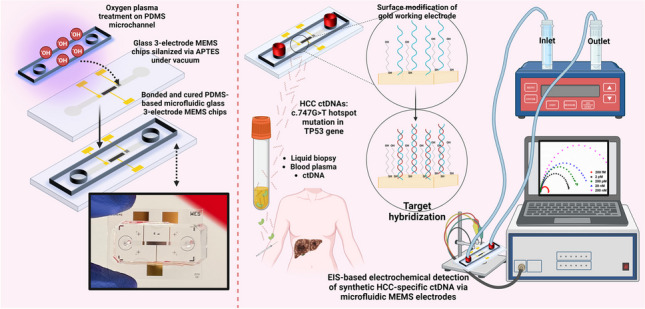

**Supplementary Information:**

The online version contains supplementary material available at 10.1007/s00604-024-06315-2.

## Introduction

Global cancer statistics has reported 9.6 million cancer related deaths in 2018, and the numbers are estimated to increase with the expected value of 30 million in 20 years [1]. Among all cancer types, lung, breast, stomach, and liver cancers are of main ones causing death. Hepatocellular cancer (HCC), known as the primary liver cancer, is the fifth most common cancer and has the second place in fatality according to the latest reports of World Health Organization (WHO). Almost a million people are diagnosed with HCC every year with 600,000 deaths, which are estimated to reach 1.5 million in 2040 [1]. Among main risk factors causing HCC are chronic hepatitis B (HBV) and hepatitis C (HCV) infection, alcohol consumption, aflatoxin, and obesity [2]. The pathological profiling of HCC, which is heterogeneous and rather complex, can be done by examining the biopsy samples. The major drawback of this process is that it is invasive and difficult to perform on a regular basis. Moreover, the biopsy sample taken contains limited information regarding the tumor and does not reflect its heterogeneity. Alternatively, liquid biopsy has emerged as a new diagnostic method. The concept is based on taking fluid samples from body such as blood or urine and focusing on the analysis of circulating tumor cells (CTCs) and circulating tumor DNAs (ctDNAs) within to help diagnosis and generate information regarding the cancer [3, 4]. Comparing the two biomarkers mentioned, the success rate in CTC isolation is low and the antibody used in their detection, called epithelial cell adhesion molecule (EpCAM), yield positive in only 20% of the HCC patients [5]. Cell-free fragments of DNAs released into the bloodstream by apoptotic and necrotic cells, in short called cell-free DNAs (cfDNAs), specifically those shed by the tumor cells (ctDNAs) can contain information regarding cancer characteristics and their amount are correlated with tumor staging and prognosis [4]. ctDNAs contain approximately 170 base pairs (some as short as 30 base pairs) and have very short half-life of 16 min to 2.5 h [6]. The cancer related characteristics they carry can be single-nucleotide mutations, methylation changes, or cancer-associated viral sequences. Alpha-fetoprotein (AFP), which is currently used in the HCC diagnosis, has limited diagnostic value due to its low sensitivity of 50% [7]. Studies conducted have proved that liquid biopsy–based methods are rather efficient in early diagnosis and prognosis of HCC [8]. It is reported that high diagnostic value of ctDNA in HCC shows good clinical correlation compared to other plasma markers [8]. As HCC has high intra-tumoral heterogeneity, it becomes especially crucial to detect some hotspot mutations in CTNNB1 genes in tumor protein 53 (TP53), catenin (cadherin-associated protein) beta-1, and telomerase reverse transcriptase (TERT) in ctDNAs [9, 10]. Recent improvements in the accuracy and sensitivity of ctDNA analysis also paved the way for genotyping of somatic genomic alterations. Therefore, ctDNA-based detection mechanisms are of great importance to further enlighten this area and become pioneer works to carry this topic forrader. Thanks to the rapid developments in biosensing technology in recent years, new and promising solutions for ctDNA detection have emerged. Among biosensors, electrochemical (EC) DNA biosensors have become an important field of study and have shown significant growth, as they allow the recognition of target DNA selectively and sensitively. Particularly, the ability to develop reliable and low-cost EC sensors owing to MEMS technology makes DNA-based EC biosensors preferable for HCC-specific ctDNA detection.

There is a growing interest in the field of microfluidic biosensors. Integration of biosensors with microfluidics improves the analytical performance of the overall system by reducing the sample volume and analysis time with the additional advantage of being portable [11]. Higher sensitivities can be achieved with microfluidic biosensors since the mass transfer to the sensor surface is enhanced through flow [12]. Microfluidics allow control of fluids on sub-millimeter scale in microchannels [13–15]. With the recent developments, microfluidics has become a major part of Lab-on-a-Chip (LOC) systems where multiple operations run on a single chip. LOC concept is considered as a promising solution to meet the requirements encountered in point-of-care (POC) systems through ease of use and ability to answer with high accuracy [16]. As a result, LOC platforms are extensively employed in POC analyses including biomarker detections [17, 18].

While early structures focused on microelectronics for the sake of highly developed photolithography and glass and silicon etching, new materials are favored in the current era, such as polydimethyl siloxane (PDMS). PDMS is one of the most preferred material in microfluidic biosensors [19–21]. Main reasons for its frequent use are its high biocompatibility, inexpensive, and non-toxic nature and exceptional fidelity of reproduction from micro-scale molding [22]. Other advantages include its ability to work well with aqueous media, high transparency, flexibility, and disallowance of non-specific adsorptions [23, 24]. As PDMS-based microfluidics began to be mainly employed in LOC systems, where a single miniaturized platform is equipped with an analytical measurement setup, on-chip DNA detection has become its foremost wielder.

Recently, development of biosensors based on DNA detection gained pace owing to their potential of allowing various applications including diagnostics [25]. DNA biosensors have been built in different detection techniques; however, specifically EC-based ones have attracted significant attention and have become the most preferred over others, such as optical because they enable cost-efficiency, relatively simpler setup, and ease of miniaturization [26, 27]. The literature is abundant in electrochemical detection of proteins, DNA, etc. as the technique provides very limit of detection (LOD) values [28, 29]. Although various biosensors can be integrated with microfluidics, electrochemical ones have been considered extensively due to miniaturization facility and integration ability, and being low cost as an established POC diagnosis in addition to its allowance to lower LODs [30]. Jang et al. employed a PDMS-based microfluidic chip to perform electrochemical immunoassay [31]. All steps were performed under flow, expediting the assay procedure compared to static ones. They used cyclic voltammetry for the enzyme–substrate reaction and obtained an LOD of 485 pg/mL. Ben-Yoav et al. established an electrochemical assay for DNA detection in a microfluidic chip with valves, for which an LOD of 1 nM was achieved [32]. Ölcer et al. used amperometry for the detection of bacteria DNA in microfluidic chips and reached LOD as low as 6 pM [25]. Another microfluidic electrochemical biosensor for bacteria DNA detection was realized by Zribi et al. allowing working with a large dynamic range from 0.1 fM to 1 pM [33].

**Fig. 1:**
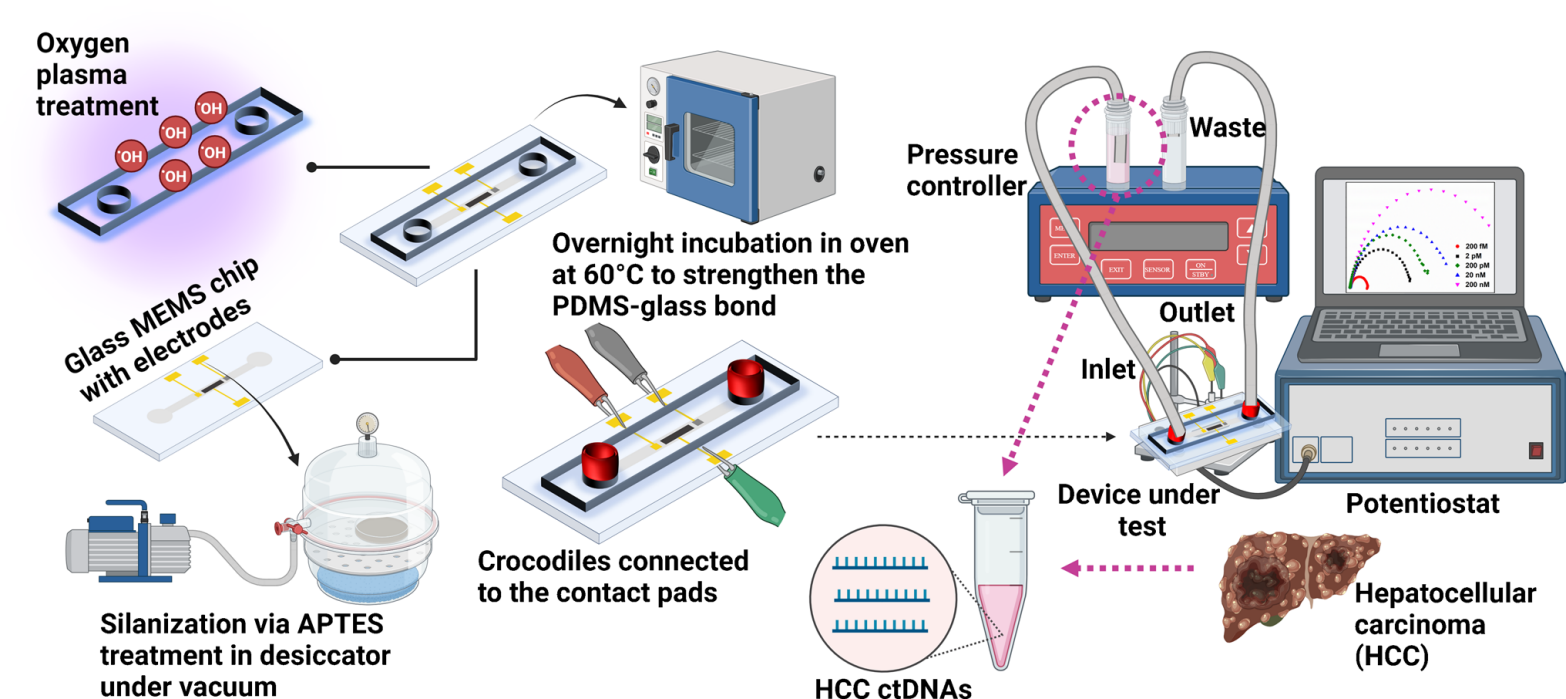
An overall scheme of the realized PDMS-based microfluidic MEMS electrochemical system for the detection of specific mutation in HCC ctDNA

This study describes the production of a PDMS microfluidic platform with MEMS fabricated three-electrode configuration consisting of Au working electrode, Pt counter electrode and Ag reference electrode, and its employment in the detection of ctDNA specific for critical mutations developed in HCC (Fig. 1). Fully complementary, fully noncomplementary and 1-base mismatched target DNA sequences were tested in the developed system. The sensor allows reaching low detection limits with high specificity. The proposed microfluidic EC HCC sensor is label-free, does not require polymerase chain reaction (PCR), and offers advantages such as low sample volume, minimum waste, ease of application, high sensitivity, and fast response time. To the best of our knowledge, this is the first time a PDMS-based microfluidic electrochemical sensor is presented to target HCC ctDNAs.

## Materials and methods

Phosphate buffered saline (PBS) (pH 7.4), blocking reagent 6-mercapto-1-hexanol (6-MHO, 99%), tris–EDTA buffer (pH 7.5 and 8.0, TE), dimethyl sulfoxide (DMSO), 2-propanol (IPA), (3-aminopropyl)triethoxysilane (APTES), and trichloro(1H,1H,2H,2H-perfluorooctyl)silane (97%, TCPFS) were purchased from Sigma Aldrich. The SYLGARD™ 184 Silicon Elastomer Kit containing poly(dimethylsiloxane) (PDMS) and elastomer crosslinker was purchased from Dow, USA.

HCC-specific critical mutations in ctDNAs require examination of certain gene regions. In this study, synthetic single-stranded DNA probes (ssDNAs) were employed, which were designed to represent the c.747G > T hotspot mutation in the TP53 gene that is frequently (30%) observed in HCC. The ssDNAs were purchased from Oligomer Biotechnology (Ankara, Turkey).

DNA sequences used in electrochemical setup are as follows:

**Table:** 

Probe Type	Sequence
Capture probe	5′-HS-C6-GTGGTACTCGCGA**A**GAGTCTATCGC-3′
Fully complementary target ctDNA (full comp)	5′-GCGATAGACTC**T**TCGCGAGTACCAC-3′
One-base-mismatched target ctDNA (1N noncomp)	5′-GCGATAGACTCGTCGCGAGTACCAC-3′
Fully noncomplementary target ctDNA (full noncomp)	5′-AACGCTTGGCAGGGATACCAGATGT-3′

c.747G > T hotspot mutation is indicated in bold, mismatched base in 1N noncomp and full noncomp are marked with underlines

All DNA probes were diluted with TE buffer and stored at − 20℃ as aliquots to eliminate multiple freezing-melting cycles.

Metal deposited three-electrode glass chips and silicon molds with channel patterns were fabricated in clean room facility of METU MEMS Center, Ankara, Turkey. Metal layer thicknesses were measured using a Dektak^®^ 8 Programmable Surface Profiler Measuring System in the clean room facility of the METU MEMS Center, oxygen plasma process was conducted with ATTO Low Pressure Plasma System, Diener Electronic, Germany. Microfluidic studies were conducted with Fluidic Flow Control System MFCS™ Series, Fluigent, Germany. Electrochemical impedance spectroscopy (EIS) was performed using Autolab PGSTAT 128N with frequency response analyzer (FRA) module, Metrohm, Switzerland. Curve fitting processes were completed in Nova 2.1.6 software, Metrohm, Switzerland.

### Design and fabrication of MEMS electrodes and microfluidic chips

**Fig. 2:**
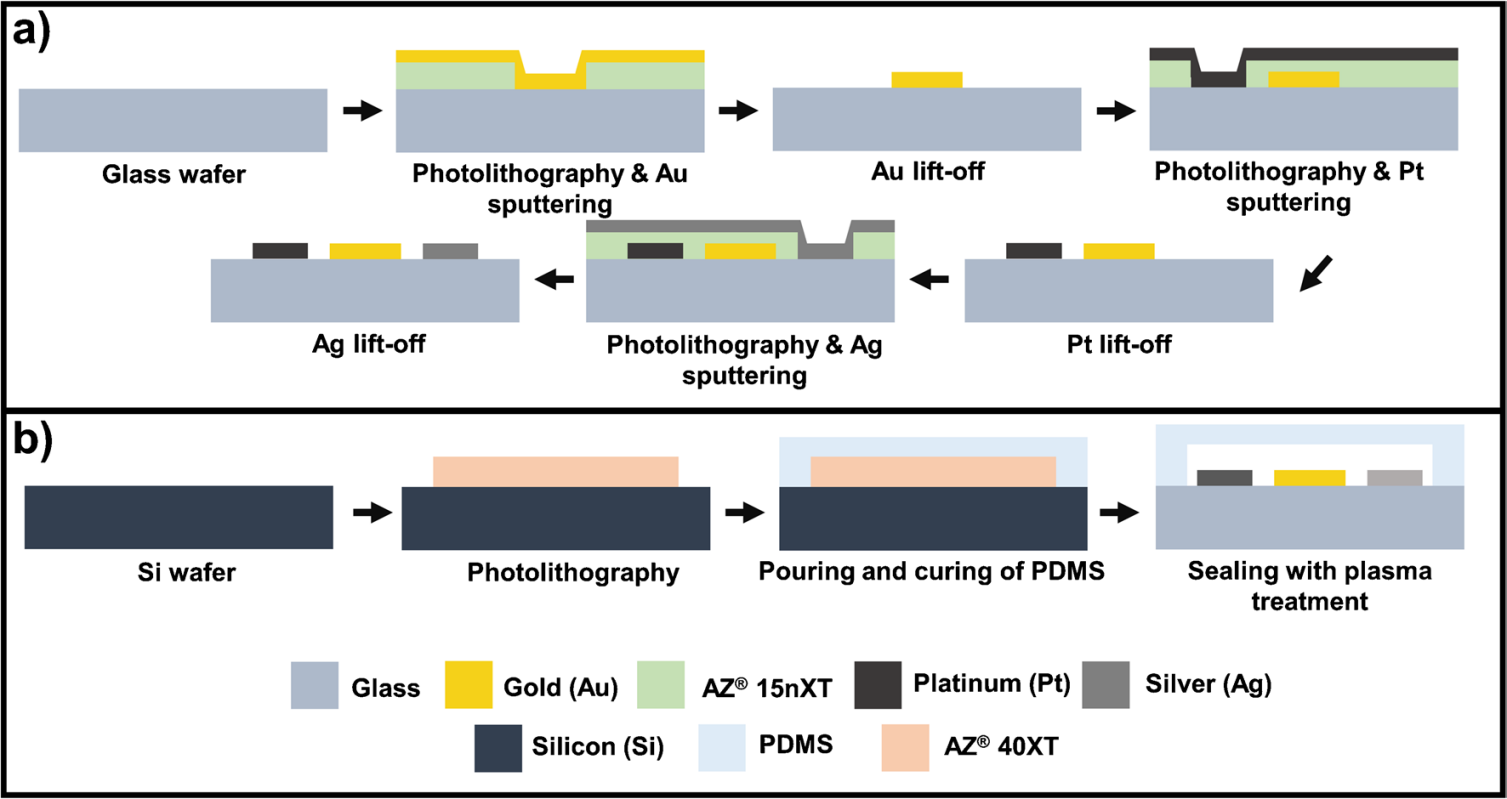
**a** Fabrication flow of Au-Pt–Ag three-electrode systems on the glass substrate. **b** Fabrication flow of PDMS microchannel structures and sealing to reach a microfluidic electrochemical biosensing platform

The electrochemical sensor platform consists of a glass on which planar electrodes and electrical connections/pads are shaped, and a PDMS microchannel. These two parts are bonded together to complete the proposed platform. Electrochemical sensors are in a three-electrode configuration that includes an Au working electrode (WE), a Pt counter electrode (CE), and an Ag reference electrode (RE). The fabrication flow of the three-material electrodes mainly consists of sequential photolithographic, sputtering, and lift-off processes which are schematically summarized in Fig. 2a. The fabrication of the biosensor is based on MEMS technology, which is applied onto 6" glass wafers used as substrates. Ti is deposited on the glass before evaporation of all electrode metals involved to ensure better adhesion. Ti as an adhesive layer was deliberately chosen instead of the chromium because the adhesive chromium layer suffers severe corrosion during electrochemical processes [34]. Initially, the Au working electrode (500 × 900 µm^2^) layer (500 nm) with Ti as the adherent layer (50 nm) is patterned by a lift-off process. The second step is the definition of the Pt counter electrode (5000 × 900 µm^2^). A Pt thin film of 500 nm thickness with Ti of 50 nm (adhesive layer) is patterned with lift-off. After this step, the geometry of the Ag reference electrode (500 × 900 µm^2^) is defined by the third photolithographic process. After that, deposited 50/500 nm Ti/Ag layers are patterned with lift-off.

Fabricated Au-Pt–Ag three-electrode electrochemical platforms are integrated with PDMS microchannel with single inlet–outlet to attain a microfluidic electrochemical device. Figure 2b shows the fabrication flow of the PDMS microchannel and bonding to the glass sensor side. Initially, the 4" silicon master is patterned with 40 µm thick to 1300 µm wide AZ^®^ 40 XT photoresist. To create microchannels, PDMS elastomer base and curing agent (weight ratio 10:1) are mixed vigorously using a mechanical stirrer (1400 rpm, 5 min) and placed in a vacuum desiccator for approximately 45 min. Degassed PDMS is then poured onto the Si master attached in a petri dish, and the entire assembly is placed in the oven at 60 °C for 5 h to completely cure the PDMS. The bonding of the PDMS microchannels peeled from Si mold and the glass side is achieved by silanization of glass chips with APTES overnight in a vacuumed desiccator and introducing hydroxyl groups on PDMS microchannels via oxygen plasma (40W, 1 min). After the plasma treatment, the PDMS microchannels were immediately brought together with silanized glass chips and incubated in oven for overnight at 60 °C to achieve irreversible bonding between the class chips and the PDMS microchannels.

### Surface preparation of the sensor system

**Fig. 3:**
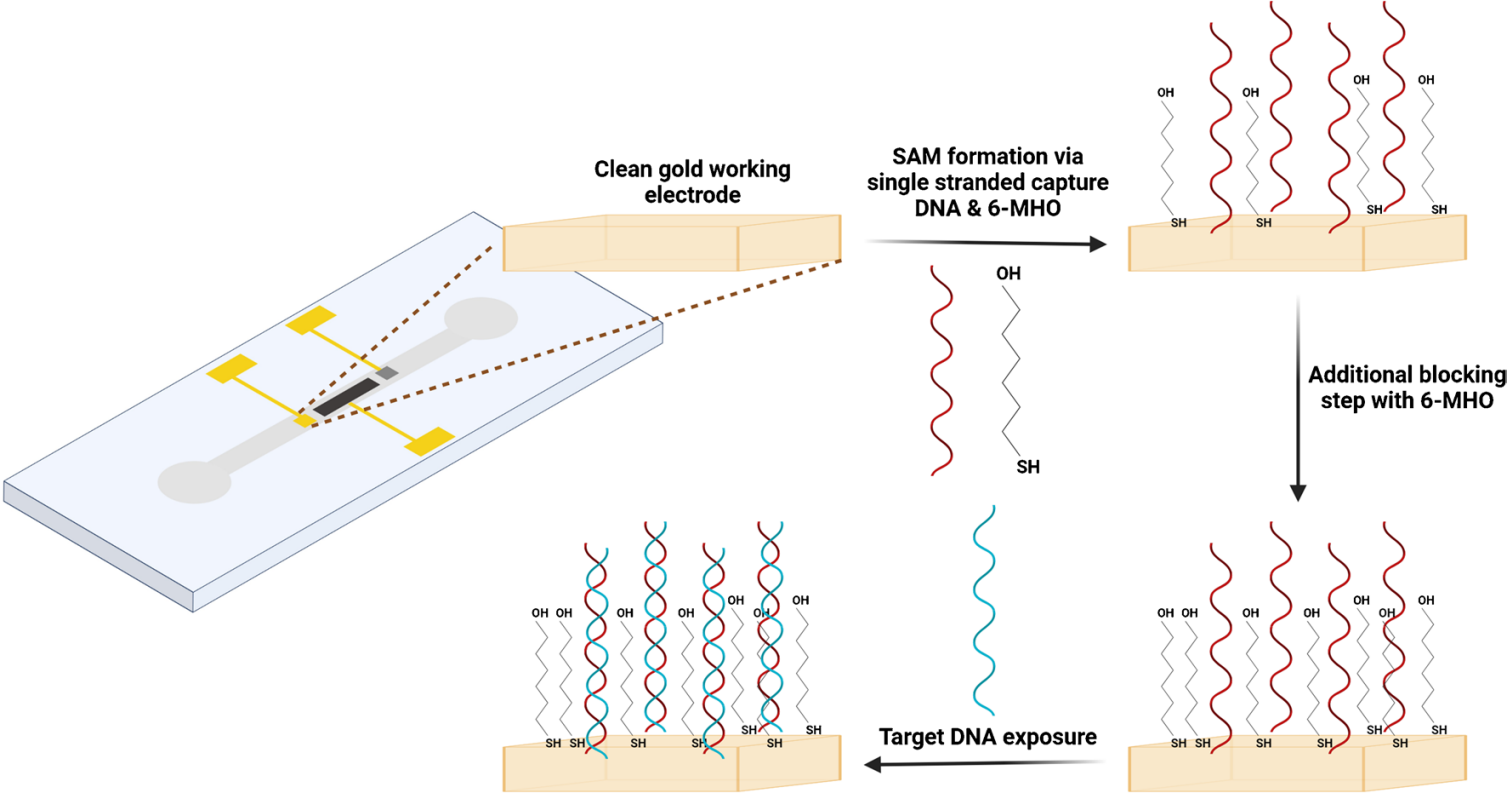
Schematic representation of the Au WE surface modification and hybridization steps

Prior to all electrochemical measurements, optimization studies were conducted for the determination of the appropriate capture probe concentration and immobilization time (Figure S2,S3). Before any surface modification, the microfluidic chips were cleaned with ethanol, IPA, and deionized (DI) water under flow, followed by running of TE buffer for electrode conditioning. The capture DNA probes were immobilized on the cleaned gold WE through their thiol (-SH) groups (Fig. 3) under flow by incubating the DNA solution (2 µM) containing 50 mM NaCl and 1 mM 6-MHO for 1 h. After washing the surfaces with PBS and DI water in the given order under flow to eliminate the unbound ssDNAs, blocking was performed by incubating the channel with 30 µM 6-MHO for 30 min. For the blocking process, 6-MHO was dissolved in DI water instead of ethanol to prevent any damage to DNAs. This additional blocking was performed to further cover the free areas left on WE to enhance the quality of the surface modification and prevent non-specific surface attachments. Next, PBS and DI water ran again through the channels under flow to remove any non-specific attachment. During all steps, the flow rate was kept constant at 30 µl/min and temperature was maintained at room temperature (RT).

### Electrochemical validation studies with PDMS microfluidic chips

Prior to all electrochemical measurements, optimization studies were conducted for the determination of DNA hybridization time (Figure S4). Target DNAs at different concentrations between the range of 2–200 fM were incubated in the channels (Fig. 3) for 1 h at RT with a flow rate of 30 µl/min. Then, the chips were rinsed with PBS and DI water in the given order by running the liquids through the channels. After this step, electrolyte 1 mM K[Fe(CN)_6_]^3−/4−^ containing 0.01 M KCl was introduced to the channels and electrochemical measurements were conducted using electrochemical impedance spectroscopy (EIS). The spectra were recorded before (ssDNA immobilized and blocking applied) and after target ctDNA exposures and surface impedance changes were calculated based on hybridization events.

## Results and discussion

### PDMS-based microfluidic electrochemical sensors

**Fig. 4:**
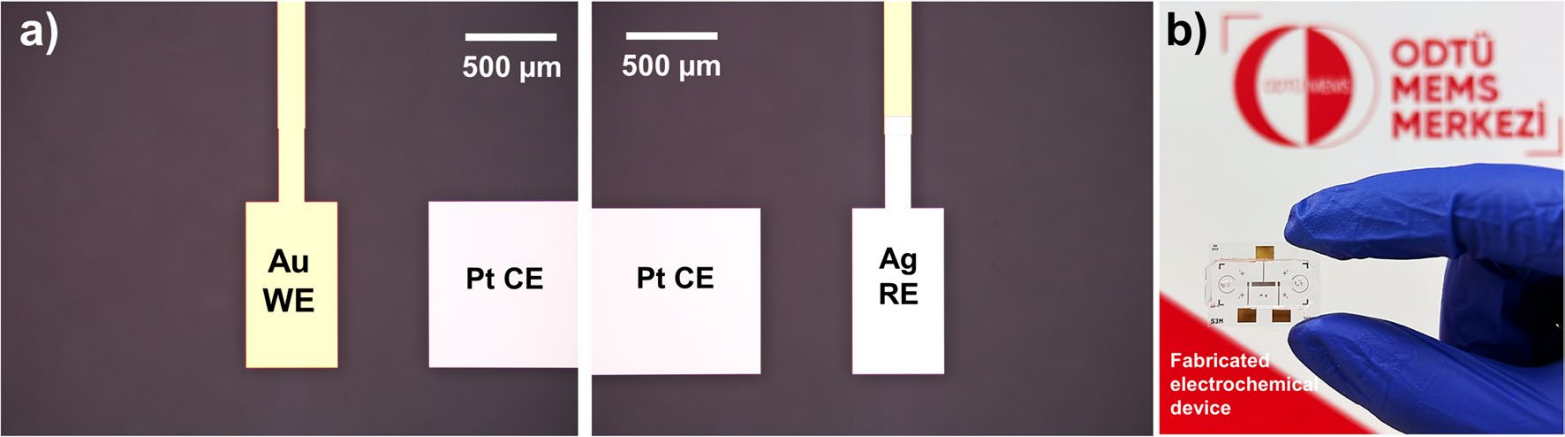
**a** Microscope images of the fabricated electrodes on the glass substrate. **b** Real image of the PDMS microchannel bonded to three-electrode glass sensor

Fabrication flow of Au-Pt–Ag three-electrode systems on the glass substrate was successfully realized (Figure S1). The thickness of each metal layer was measured after the corresponding lift-off step was completed during MEMS fabrication of the devices. In measurements made using the Dektak^®^ 8 Programmable Surface Profiler Measurement System, the metal thickness of each electrode (Ti/Au, Ti/Pt and Ti/Ag) was determined as 550 nm ± 20 nm. Microscope images of the fabricated electrodes are given in Fig. 4a. During the bonding of PDMS microchannels with glass chips, initially the effect of plasma treatment to the electrodes was tested and resulted in an unsuccessful attempt due to the damage mainly caused to silver electrode by the intense oxygen exposure. Therefore, silanization of the glass chips via APTES was adopted as an alternative to the literature [35, 36] and plasma treatment was done only to PDMS microchannels. Following the surface modifications, by adding a few droplets of DI water on the chip, PDMS microchannels can easily be adjusted to sit directly on the fabricated electrodes on the glass. The prepared chips were then let to stay on hot plate set to 60 °C for a few minutes until the dropped water evaporates. Afterwards, chips were incubated overnight in oven at 60 °C to complete irreversible bonding.

### Validation studies with HCC hotspot mutation specific ctDNA probes

Validation of the developed microfluidic EC sensors was conducted by HCC specific ctDNA probes containing c.747G>T hotspot mutation in the TP53 gene. The ctDNA concentrations varied between 2 fM to 200 fM. EIS was adopted during the electrochemical measurements before and after hybridization of the probes with the target. EIS is a powerful technique commonly employed in the EC detection of biorecognition [37]. It is a sensitive tool that enable label-free detection with high signal-to-noise ratio suitable for in situ analysis [38]. Obtained Nyquist plots in impedance spectra were fitted to an equivalent circuit model describing the electrical properties and behaviours at the electrochemical interface. Equivalent circuit models are formed by connecting resistors, capacitors, and other circuit elements in parallel or in series and are employed to represent electrochemical processes. One of the most exercised equivalent circuit models to represent the impedance of an electrochemical biosensor with a three-electrode configuration is the Randles equivalent circuit. It consists of electrical resistance of the analyte (*R*_*s*_), charge transfer characteristics at the electrode/electrolyte interface (*R*_ct_), double layer capacitance at the electrode/electrolyte interface (*C*_dl_), and an optional element called the Warburg impedance (*Z*_*w*_) describing the diffusion processes of the reactants. In case of resulting Nyquist plots that lack a diffusion-controlled region, *Z*_*w*_ can be omitted. The measured experimental data are represented with a Nyquist plot where imaginary impedance (*Im(Z)*) versus real impedance (*Re(Z)*) is plotted for each frequency [38, 39]. The measured impedance data in the form of Nyquist plot is then fitted into equivalent circuit model to analyse the electrical properties of the sample.

**Fig. 5:**
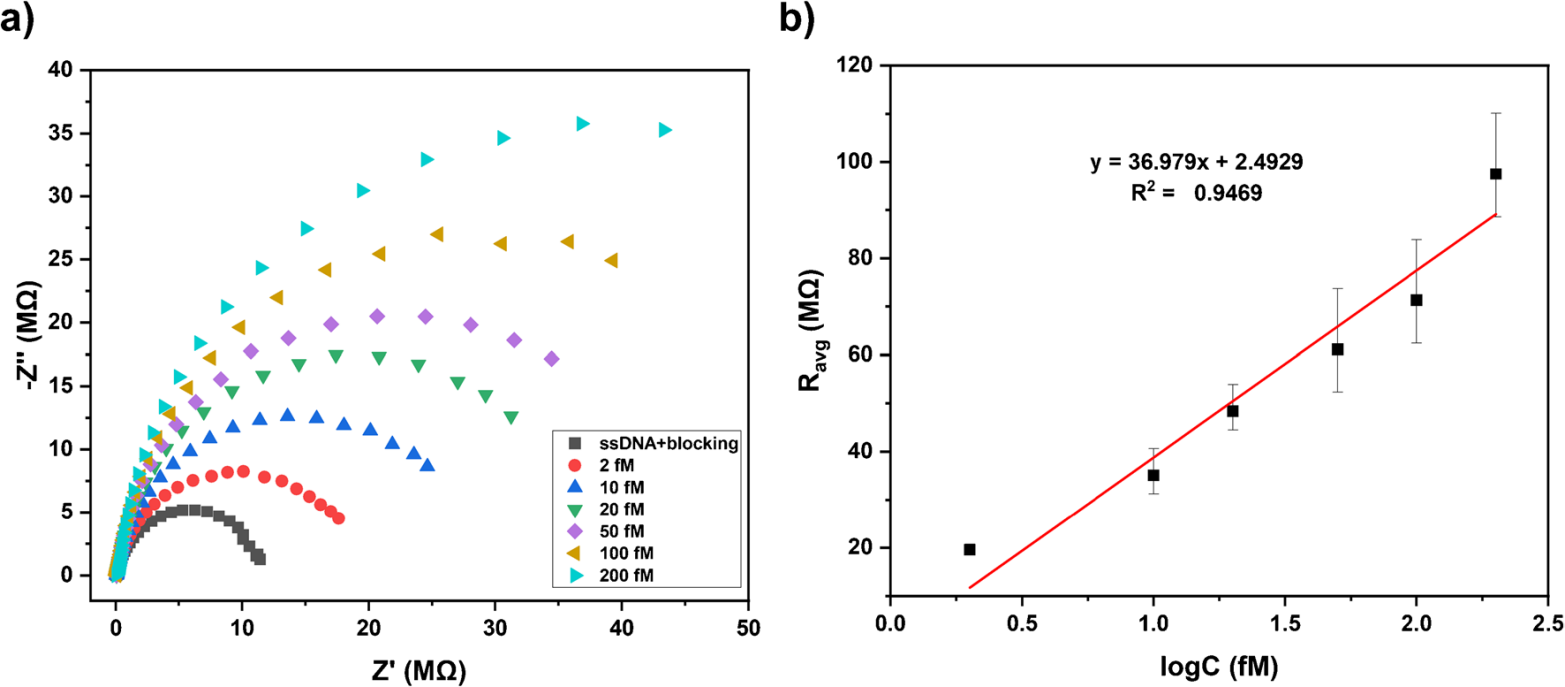
**a** Nyquist plots for different concentrations of target ctDNA. **b** Calibration curve for the studied concentrations

The Nyquist plots obtained in this study were in semi-spherical shape, representing the simplest example of Randles circuit where a resistor and capacitor were connected in parallel. In this case the model includes a solution resistance and a constant phase element (CPE) for the Faradic impedance resulting from the electrochemical process. Here, the Faradic impedance is only the charge transfer resistance (*R*_ct_). The Warburg impedance (*Z*_*w*_), which usually included due to mass transfer to the electrode surface, was excluded as there was merely a negligible effect by the addition of *Z*_*w*_. Since the Warburg element is associate with the tail of the Nyquist plot corresponding to diffusion-controlled region [37], its omission was ineffective owing to the complete semi-spherical nature of the plots obtained without any tail. EIS responses after each modification and hybridization steps can be found in Fig. S4. The Nyquist plots obtained for the studied concentrations are provided in Fig. 5a. The calibration curve drawn upon these results yielded an *R*^2^ value of 0.9496, showing very high linearity (Fig. 5b). This linearity, however, can be enhanced further by filling the concentration gaps and adding more to the further studies. Regression analysis was carried out using the data obtained, from which the value was calculated from the regression analysis of the calibration curve data. The well-known formula of $3.3\sigma /S$ was used where *σ* is the standard deviation about the regression line for the entire data and *S* is the concentration coefficient from the regression statistics. The LOD was calculated as 24.1 fM. The ctDNA concentration in plasma of HCC patients is reported to be 28.2 ng/mL (median), ranging between 9.8 and 487.1 ng/mL [40]. The synthetic ctDNA sequence used in this study contains 25 bases and from the calculations its weight is 7705.97 Da (g/mol). The lowest concentration tested is 2 fM, corresponding to 3.853 ng/mL, falling within the range of ctDNA concentration in HCC patients. Therefore, the sensor can respond to very low and appropriate concentrations as is without any pre-enrichment steps such as PCR.

**Fig. 6:**
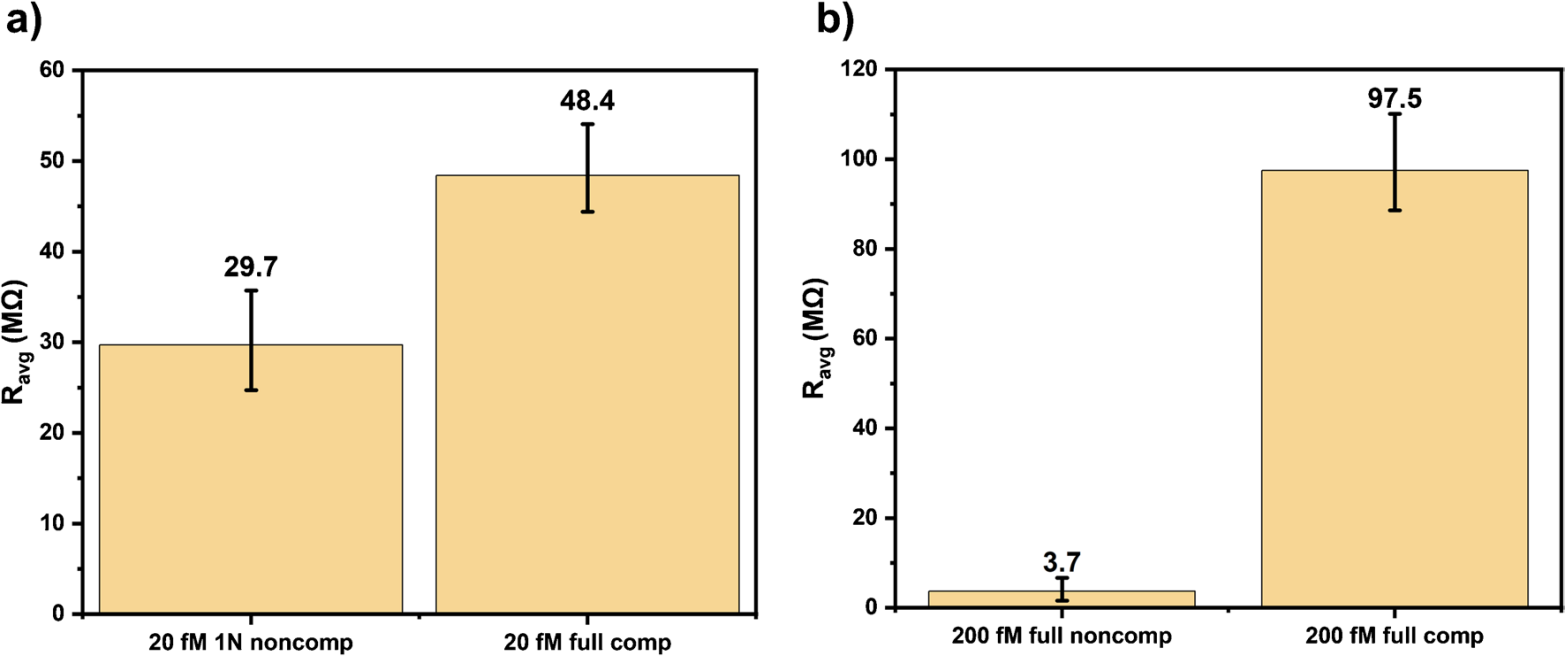
Normalized impedance responses of the chips for the **a** detection limit concentration (20 fM) of 1-base mismatched (1N noncomp) and full comp targets, **b** highest tested concentration (200 fM) of full noncomp and full comp targets

The specificity of the system was checked by studying the impedance differences occurred upon exposure of the modified chips to the full noncomp and 1N noncomp target DNAs (Fig. 6a, b). For the 1-base mismatched target (1N noncomp), 20 fM was studied to reveal the sensor’s response for non-target sequences down to 1-base at develop platform’s detection limit. A difference of 1.6 folds was obtained between 1N noncomp and full comp impedance responses (Fig. 6a). A similar study was applied also for full noncomplementary sequences (full noncomp) at highest tested concentration of 200 fM to examine the sensor’s response for abundant fully non-specific sequences. A difference of 26 folds was obtained between full noncomp and full comp impedance responses (Fig. 6b), showing the high specificity of the developed system.

The number of studies where liquid biopsy biomarker detection meets microfluidics are increasing in the literature. One example is by Khaksari et al. whose study focuses on a microfluidic EC aptasensors to detect A549 cells as CTC model of non-small-cell lung cancer (NSCLC) [41]. They reported an LOD value of 14 cells/mL. They used differential pulse voltammetry (DPV) during their electrochemical measurements and the data were collected both on- and off-chip. Similar to our study, merely single-stranded thiolated oligonucleotides were used rather than nanoparticles, graphene, or complex structures, which greatly simplifies the sensor. One major difference is that only the microfluidic chamber was manufactured, and a commercially available screen-printed electrode was used. Ölcer et al. developed a microfluidic biosensor to detect bacteria using DNA [25]. They performed amperometric measurement, added enzyme-modified gold nanoparticles to their system, and obtained an LOD value of 6 pM. Although well-known binding mechanisms were employed, additional particles embedded into the sensor increase the complexity. Zribi et al. recorded an LOD value of 0.7 fM on their microfluidic EC sensor for the detection of *Mycobacterium tuberculosis* using DNA [33]. They ran the experiments at high flow of 150 µl/min, shortening the time. Another crucial point in the study was that by preferring high flow, thin depletion layer at the sensor surface increased the capture rate of DNA (1 DNA/sec) compared to static regime, where the rate was 0.02 molecule/sec. One of our aims was as well to increase the number of attached DNAs on the electrode surface by integrating a flow mechanism to the chips. Based on our experiences with previous static conditions, more DNA was attached to the surface in shorter time when fluidics were integrated to the system. Lim et al. used a two-electrode chip to build a microfluidic biochip based on aptamer to detect thrombin using EIS [42]. The biochip had an LOD of 0.1 ng/mL. One drawback of the study was that the gold-thiol interaction was performed for 24 h. Instead of such dramatic preparation periods, we aimed to shorten these steps. During the optimization studies with MEMS fabricated static three-electrode chips, it was found that immobilization time as short as 1 h resulted in a certain surface coverage (Figure S3). As one of the aims of microfluidic sensors is to reduce the preparation and sensing times, the proposed system well agrees with this purpose. Vaselinovic et al. focused on nanostructuring the electrode surface to achieve lower LODs by providing higher surface coverage of capture probes [43]. It was found in the study that electro-grafting reduced the incubation time of capture DNA probes into the porous electrodes down to 10 min, meeting the very essence of time-efficiency in terms of preparation steps. To achieve this, they employed gold nanoparticle multielectrode arrays to detect DNAs with cancer-related genes. This technique could be used to further enhance the setup proposed in our study, where electrode structures are planned to contain micropillars.

Apart from shortening the preparation and analysis times of the sensor, the presented study focused on the aims of reducing the sample volumes and presenting a label-free design without complex preparation steps. While some nanoparticles and composites may offer enhancements such as further lower LODs, the proposed system distinguishes itself for its ease of setup and device structure. It achieves a substantially low LOD in the fM range and demonstrates a concise immobilization and analysis time of 2 h in total (including surface modification and hybridization). Additionally, it operates with a modest sample volume of 2 mL and facilitates label-free detection of HCC ctDNAs. Notably, the presented system achieves these accomplishments without the necessity for intricate composites or device structures, thereby optimizing experimental procedures.

## Conclusions

In this study, glass three-electrode MEMS chips were fabricated and PDMS microchannels were produced to establish a PDMS-based EC microfluidic sensor for the detection of c.747G > T hotspot mutations in TP53 gene of HCC ctDNAs. To the best of our knowledge, this is the first time a microfluidic EC sensor is studied to detect HCC specific ctDNA. The system provides LOD of 24.1 fM with high specificity. As this is the first study to validate the proposed system to specifically aiming to analyze c.747G > T hotspot mutations in TP53 gene of HCC, a concentration range of 2–200 fM target ctDNA concentrations were selected, aiming to fall into the reported plasma ctDNA range of 9.8 to 487.1 ng/mL**,** and corresponding EC responses were examined. The proposed sensor does not require any pre-enrichment steps, such as PCR, and the LOD obtained corresponds to approximately 40 ng/mL, which is within the ctDNA concentration range in HCC patients [40]. As the system has proved itself in terms of recognition of the hybridization event with high sensitivity and specificity, further studies should focus on the ultimate goal which is to optimize the proposed sensor with plasma and test the system with real ctDNA samples from HCC patients.

### Supplementary Information

Below is the link to the electronic supplementary material.

Supplementary file1 (DOCX 9298 KB)

## Data Availability

The data that support the findings of this study are available from the corresponding author upon reasonable request.
