# Supplementary material for: Pre-enrichment-free detection of hepatocellular carcinoma-specific ctDNA via PDMS and MEMS-based microfluidic sensor
Source: Mikrochim Acta. 2024 Apr 2;191(5):229. doi: 10.1007/s00604-024-06315-2 (PMC10987365; doi:10.1007/s00604-024-06315-2)
Supplement: Supplementary file 1 — Supplementary file1 (DOCX 9298 KB) [file 604_2024_6315_MOESM1_ESM.docx]

Supporting Information

For

Pre-Enrichment-Free Detection of Hepatocellular Carcinoma-Specific ctDNA via PDMS and MEMS-based Microfluidic Sensor

Zeynep Çağlayan Arslan^a,c^, Meltem Okan^b,c^, Haluk Külah^a,b,c*^

a Department of Electrical and Electronics Engineering, METU, Ankara, Turkey

b Department of Micro and Nanotechnology, METU, Ankara, Turkey

c METU MEMS Research and Application Center, Ankara, Turkey

⁎Corresponding author.

*E-mail address:* [kulah@metu.edu.tr](mailto:kulah@metu.edu.tr)

Electrodes were characterized with Scanning Electron Microscopy (SEM) (Regulus 8230, Hitachi, Japan) images and Energy Dispersive X-ray (EDX) (Ultim^®^ Max 100, Oxford Instruments, UK) in the clean room facility of METU MEMS Center, Ankara, Turkey (Figure S1).

**Figure S1.** SEM images and EDX spectra of Au, Ag and Pt electrodes on finalized MEMS chips.

Optimization studies were conducted on MEMS-fabricated static 3-electrode glass chips for the concentration of ssDNA probes, immobilization time, and hybridization time. A decrease in current indicates greater surface coverage. Consequently, a concentration of 2 µM was determined as the optimal concentration for the capture probe (Figure S2). Subsequently, while maintaining the concentration at 2 µM, the immobilization time of the capture probes was optimized. Immobilization time of 1-hour yielded the highest resistance response and was thus selected as the optimal time (see Figure S3). As the current responses obtained for hybridization times of 1, 1.5, and 2 hours were similar (refer to Figure S4), a hybridization time of 1 hour was chosen and kept constant in all experiments to minimize the total modification-sensing time.


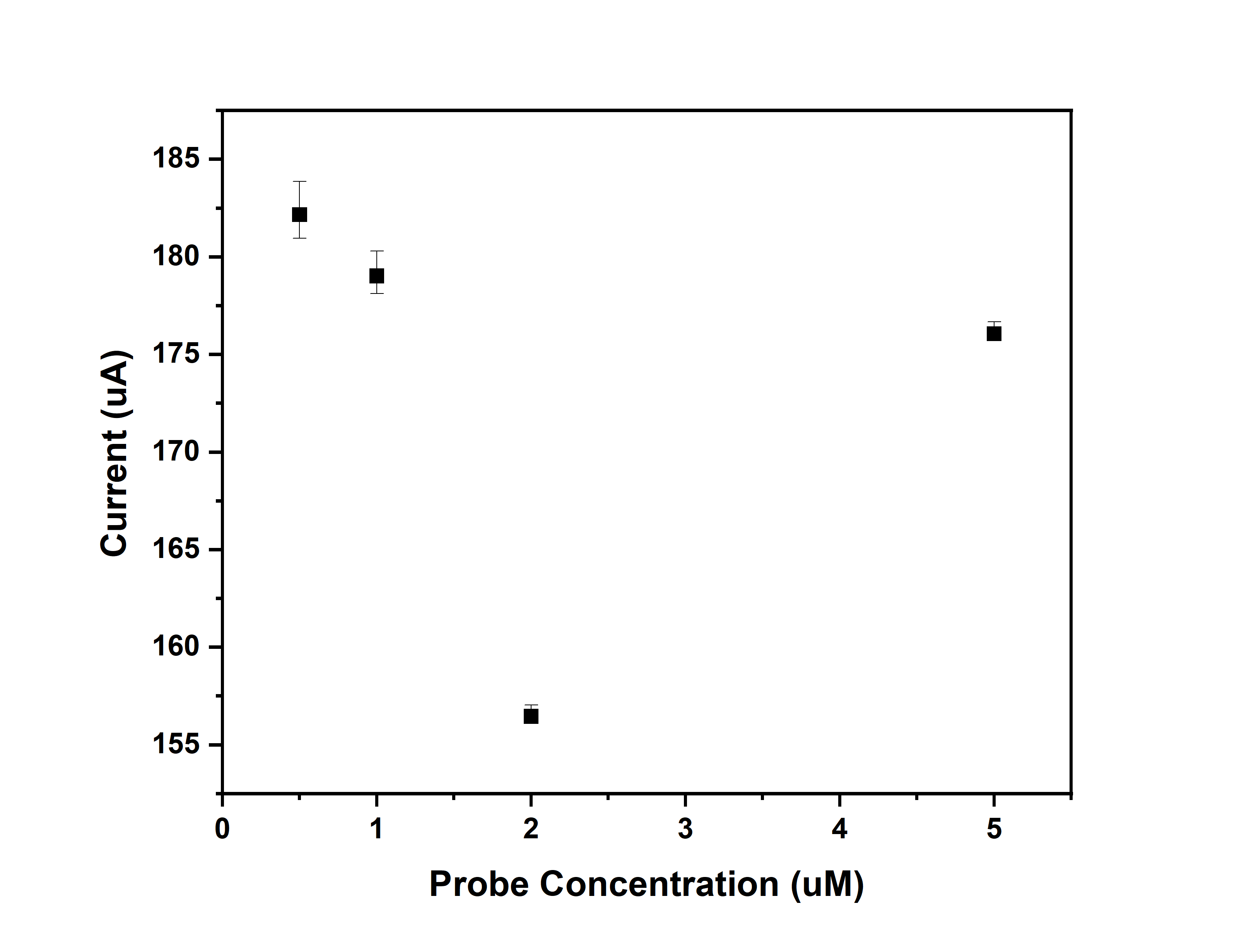


**Figure S2.** ssDNA probe concentration optimization.


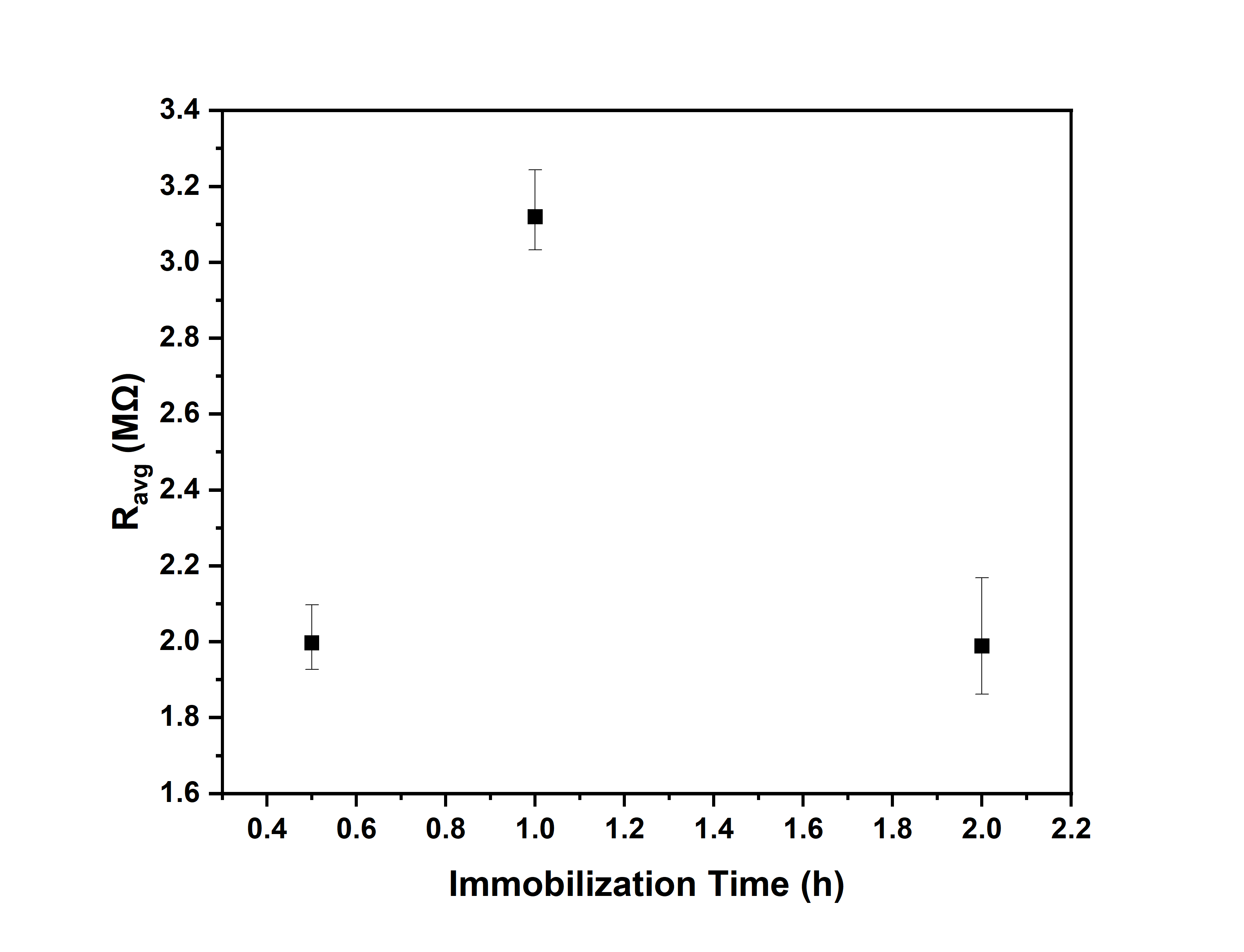


**Figure S3.** ssDNA immobilization time optimization.


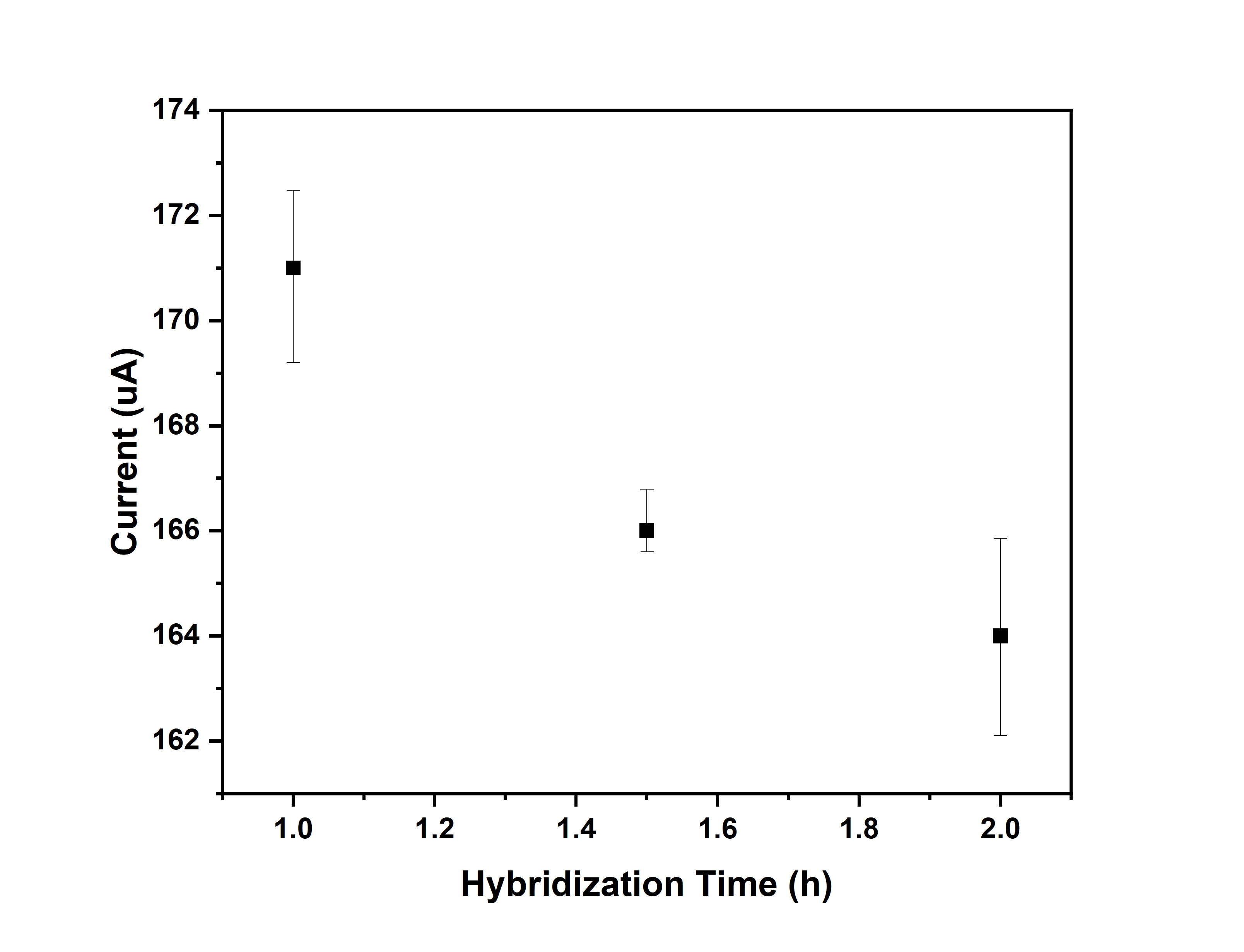


**Figure S4.** Hybridization time optimization.


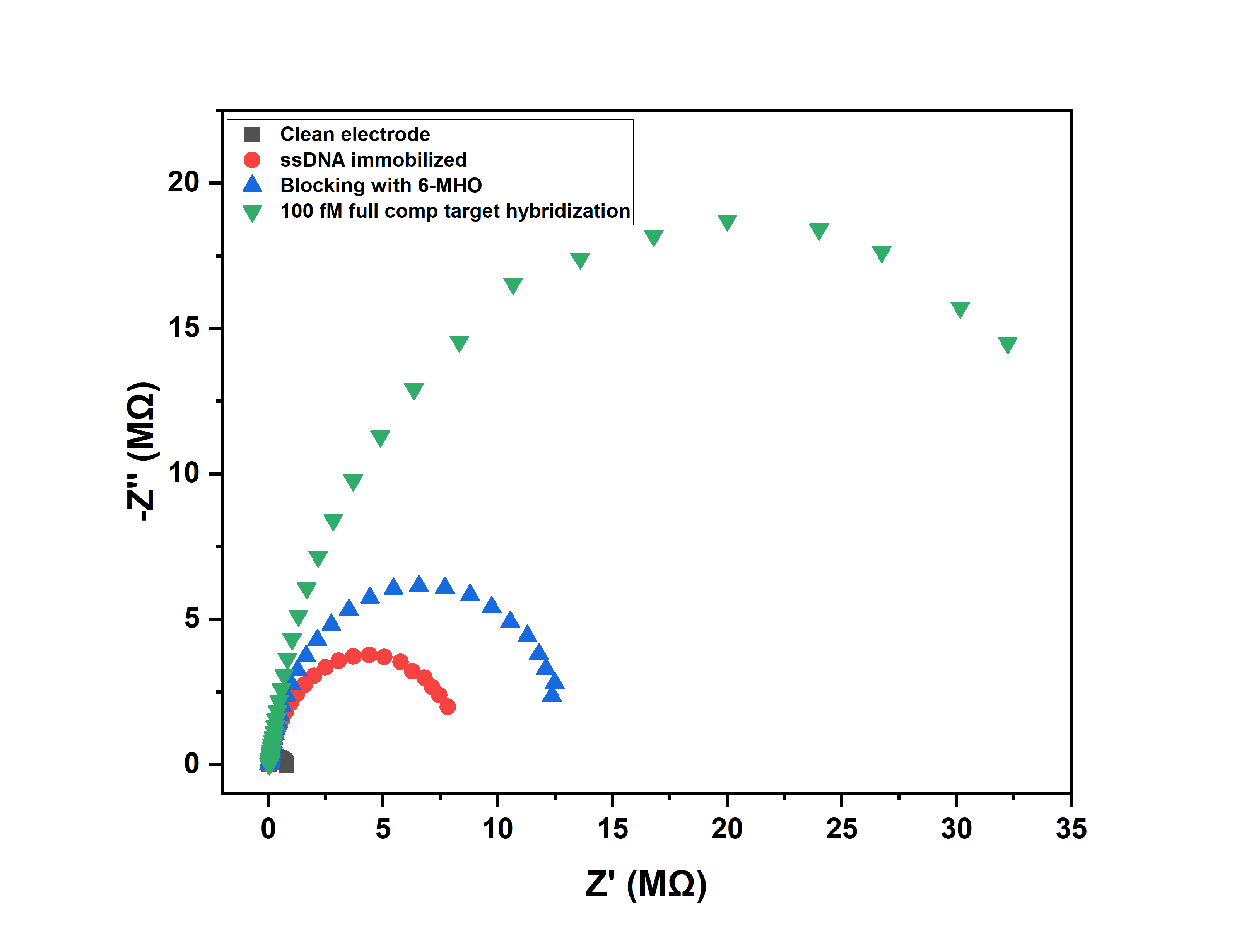


**Figure S5.** Step-by-step EIS response of the chip after each modification and hybridization steps.
